# Muscle Dysmorphia and its Associated Psychological Features in Three Groups of Recreational Athletes

**DOI:** 10.1038/s41598-018-27176-9

**Published:** 2018-06-11

**Authors:** Silvia Cerea, Gioia Bottesi, Quirico F. Pacelli, Antonio Paoli, Marta Ghisi

**Affiliations:** 1 Department of General Psychology, University of Padova, via Venezia 8, 35131 Padova, Italy; 2 Department of Biomedical Sciences, University of Padova, via Ugo Bassi 58/b, 35131 Padova, Italy

## Abstract

Muscle Dysmorphia (MD) is a psychological disorder characterized by the preoccupation with the idea that one’s body is not lean and muscular. The current study aimed to explore MD behaviours and symptoms in three groups of recreational athletes: bodybuilders (BB; *n* = 42), strength athletes (SA; *n* = 61), and fitness practitioners (FP; *n* = 22). Furthermore, we assessed MD-related psychological features as well as possible psychological predictors of MD among groups. Results highlighted that the BB group reported more beliefs about being smaller and weaker than desired compared to the other groups, whereas individuals in the SA group reported setting higher standards for themselves than the FP group. Lastly, orthorexia nervosa and social anxiety symptoms emerged as predictors of MD symptoms in the BB group. Taken together, our findings suggest that individuals in the BB group are characterized by more MD general symptomatology than those in the other groups; furthermore, only orthorexia nervosa and social anxiety may play a specific role in predicting MD general symptoms in bodybuilders.

## Introduction

Muscle Dysmorphia (MD) is a subtype of Body Dysmorphic Disorder (BDD) characterized by the preoccupation with the idea that one’s body is not sufficiently lean and muscular^1–3^. Individuals with MD perceive themselves as small and weak even if they look normal or very muscular^2^. As a consequence, individuals with MD engage in behaviours aimed at achieving the desired lean and muscular physique^2^; these behaviours are compulsive and are comprised of excessive exercise and rigid diet, excessive use of dietary supplements and, sometimes, may also include the use of anabolic-androgenic steroids (AAS)^2,4–6^. Individuals with MD frequently avoid important social or occupational activities because of the compulsive need to maintain their excessive exercise and rigid diet^2,4^.

MD affects mostly men^2^, and its prevalence rates vary significantly depending on the sample population; in particular, athletes involved in resistance training may be at increased risk for MD development compared to other athletes and non-trainers^2,7^. However, a generalization for all athletes involved in resistance training activities does not appear to be justified. A discrepancy of MD prevalence and features among subgroups within the resistance training community exists and is dependent on the goals of the weight training activity^8^. Indeed, athletes who engage in appearance-related resistance training (e.g., bodybuilders) may be at increased risk for MD development than athletes involved in resistance training to improve strength (e.g., weightlifters)^8^. In fact, many studies have underlined that bodybuilders display higher MD prevalence rates and more MD features than other resistance training athletes^2,8–11^ with prevalence rates ranging from 3.4%^9^ to 53.6%^12^ within this population. Therefore, differences in goals characterizing bodybuilders and other resistance training athletes may influence the prevalence and the manifestation of MD^13^. Although bodybuilders are considered at increased risk for developing MD^2,7,8^, the study by Pickett *et al*.^14^ reported that competitive bodybuilders did not differ from other resistance training athletes with respect to the self-evaluation of their body image; furthermore, both groups reported higher positive self-evaluation of their body than physically active controls. Moreover, both competitive bodybuilders and resistance training athletes were more satisfied about their upper torso muscles and general muscle tone than physically active controls. Finally, competitive bodybuilders reported higher levels of social self-esteem than both resistance training athletes and athletically active controls^14^. These inconsistent findings may be due to sampling methods (e. g. different level of competition) and by the self-report measures employed to assess MD that may fail in the evaluation of the condition’s key features^13^.

Dysfunctional eating patterns, as well as psychological features such as perfectionism, self-esteem, and social anxiety, may play a key role in the development and maintenance of MD^4,15,16^. MD shares clinical features with Eating Disorders (EDs), because of the presence of the compulsive exercise and rigid diet characterizing both disorders; accordingly, previous studies have well documented the high comorbidity between MD and EDs^5,17^, but little is known about dysfunctional eating patterns, such as Orthorexia Nervosa (ON) in individuals with MD. ON is defined as an obsession for healthy food and differs from EDs since dysfunctional eating patterns are expressed in a “qualitative” way rather than in a “quantitative” one^5,18^. Indeed, in order to obtain the desired lean and muscular physique, individuals with MD usually strictly control what they eat, and their food choices are focused on the healthy aspects of the food^19^. Furthermore, men’s internalization of a muscular body ideal, typical of men with MD, plays a significant role in relation to ON. The internalization of a muscular body ideal is related to higher scores of ON which explains why ON is common in individuals with MD^18,20^.

Perfectionism might be involved in MD development and maintenance, given that individuals with MD struggle to reach an unattainable body shape^8,16^. Perfectionism may influence the development of MD both directly and indirectly; the direct influence relies in the pursuit of the perfect body, whereas the indirect influence might be enacted through body dissatisfaction^16^. Although the association between perfectionism and MD is well-known and established^11,16^, little is known about which specific components of perfectionism are actually involved in MD. Indeed, perfectionism is a multidimensional construct composed of three dimensions (self-oriented perfectionism, other-oriented perfectionism, and socially-prescribed perfectionism)^21^ that may differentially contribute to the development and maintenance of MD.

Low self-esteem is also conceptualized as a crucial factor implicated in the development and maintenance of MD^4,15,16,22^. Individuals with MD may engage in appearance-improving behaviours (such as weightlifting, excessive exercise, rigid diet, and use of dietary supplements) to enhance their self-esteem^23^; importantly, the muscular development obtained performing these activities reinforce engagement in these activities again. Therefore, low self-esteem can provide a source of motivation for MD behavioral symptoms^23^. Furthermore, low self-esteem may contribute to the avoidance of social situations in which the body is exposed to others, and such avoidance is negatively reinforced by a temporary reduction in anxiety^4^. Consistently, individuals with MD try to avoid places and situations in which their bodies might be seen by others (e.g., beaches, swimming pools) or endure such situations with marked distress and anxiety^2,4^. Although the role of social physique anxiety has been well explored with respect to MD development^2,24^, little is known about social anxiety in MD. Moreover, despite the fact that low self-esteem is theoretically linked to MD^4,15,16,22^, previous studies examining self-esteem in individuals considered at increased risk for MD led to inconsistent results. For example, Muller *et al*.^25^ did not observe differences in self-esteem between bodybuilders/weight lifters and athletes practicing other sports (contact and non-contact sports). Even more surprisingly, Pickett *et al*.^14^ found that competitive bodybuilders had higher social self-esteem than physically active controls. Such contradictory results call for further research aimed to shed light on the role of self-esteem in individuals considered at increased risk for MD.

Despite the recent increased interest toward this disorder and its growing prevalence^26^, MD is still under-studied and uncharted. As previously reviewed, individuals with MD are markedly distressed and/or impaired by their preoccupation with their perceived weakness, which negatively impacts their quality of life^2,3^. A more in-depth understanding of MD, especially in athletes at increased risk, would contribute to the development and implementation of effective assessment, prevention, and treatment programs, thus further leading to higher knowledge and awareness about this disorder.

Therefore, the current study aimed to explore MD behaviours and symptoms in three groups of Italian athletes who trained regularly for recreational purposes: bodybuilders (BB group), strength training athletes (SA group), and fitness practitioners (FP group). In accordance with previous studies, we expected to observe more MD behaviours and symptoms in the BB group than in the other groups^25,27^.

Furthermore, we investigated MD-related psychological features such as self-esteem, perfectionism, social anxiety, and ON symptoms among these groups of athletes (BB, ST, and FP). Since bodybuilders are more likely to show MD features^25,27^, we expected to observe lower levels of self-esteem and higher levels of perfectionism, as well as more ON symptoms, in the BB group than in the other two groups. Furthermore, given the association between MD, low-self esteem, and social anxiety^2,4^, we hypothesized higher levels of social anxiety symptoms in the BB group than in the other two groups; to note, no previous study explored social anxiety among these groups of athletes (BB, SA, and FP).

Lastly, we sought to explore possible MD psychological predictors among groups. In light of (1) the putative role played by high levels of perfectionism, social anxiety, ON symptoms, and low self-esteem in the development and maintenance of MD^4,15,16^ and (2) the increased risk of MD in athletes involved in resistance training for appearance-related purposes^2,7^, we hypothesized that all the above mentioned psychological features would predict MD only in the BB group.

## Results

### Sample Description

The mean age of the total sample was 30.89 (*SD* = 8.90; range = 19–55), and the mean years of education was 15.02 (*SD* = 2.85; range = 8–21). The majority of participants reported being single (36%) and full time employed (46.4%). Groups were equivalent with respect to age (*p* = 0.06), years of education (*p* = 0.93), marital status (*p* = 0.21), and occupation (*p* = 0.35; Table 1), whereas differences emerged on BMI (*p* = 0.01). Bonferroni post hoc comparisons showed that the BMI of the SA group was higher than the BMI of the FP group (*p* = 0.01), while the BB group did not differ from either the SA (*p* = 0.06) or the FP (*p* = 0.99) groups (Table 1). To note, eight participants (6.4%) satisfied the Pope *et al*. (1997) diagnostic criteria for MD: four bodybuilders, two strength athletes, and two fitness practitioners.

**Table 1:** Comparisons among groups on age, education, marital status, occupation and Body Mass Index (BMI).

	BB group (N = 42)	SA group (N = 61)	FP group (N = 22)	*χ* ^2^ */F*	df	*η* ^2^_*p*_	*p*
Age	28.17 (8.14)	32.26 (9.08)	32.27 (8.97)	3.05	2,122	0.04	0.06
Education	15.10 (2.93)	14.92 (2.73)	15.14 (3.17)	0.07	2,122	0.001	0.93
Marital status (% single)	50	26.23	36.36	8.38	6	—	0.21
Occupation (% employed full time)	45.24	45.90	50	11.12	10	—	0.35
BMI	24.77 (2.12)	25.89 (2.36)^a^	24.23 (2.53)	5.32	2,122	0.08	0.01**

Note. BB = bodybuilders; SA = strength athletes; FP = fitness practitioners.

**p < 0.01.

^a^Significantly different to FP.

### MD Behaviours and Symptoms Among Groups

With respect to behaviours related to MD, Chi-squared analyses showed significant group differences on daily protein intake (χ^2^_6_ = 19.28; *p* = 0.004). Indeed, protein intake was >2 g proteins/kg/day only in the BB group compared to the other two groups (18 participants *vs* 6 participants in the SA group and 4 participants in the FP group). Groups also differed with respect to AAS use (χ^2^_6_ = 13.32; *p* = 0.04). More individuals in the BB group reported considering taking AAS (10 participants *vs* 4 participants in the SA group and no one in the FP group).

With respect to MD symptoms, ANOVAs showed significant group differences on the MDDI. In particular, the BB group scored significantly higher on the DFS subscale than the SA (*p* = 0.001) and the FP (*p* = 0.02) groups, whereas no difference between the SA and the FP groups emerged (*p* = 0.99). Moreover, the BB group scored significantly higher on the total score of the MDDI than the FP group (*p* = 0.02), while the SA group did not differ from either the BB (*p* = 0.21) or the FP groups (*p* = 0.49). With respect to other MDDI subscales, no differences among groups emerged (MDDI AI: *p* = 0.59; MDDI FI: *p* = 0.06). Means, standard deviations, and comparisons are reported in Table 2.

**Table 2:** Comparisons among bodybuilders (BB group), strength athletes (SA group) and fitness practitioners (FP group) on MD and related psychological and psychopathological features.

	BB group (N = 42)	SA group (N = 61)	FP group (N = 22)	*F*_(1,122)_	*η* ^*2*^_*p*_	*p*
MDDI desire for size	10.31 (4.32)^a^	7.05 (4.18)	7.14 (4.64)	7.85	0.11	0.001**
MDDI appearance intolerance	4.17 (2.94)	3.93 (3.02)	3.36 (2.84)	3.07	0.01	0.59
MDDI functional impairment	6.93 (4.20)	7.34 (3.61)	4.91 (4.51)	0.53	0.04	0.06
MDDI total score	21.40 (8.44)^b^	18.33 (7.83)	15.41 (9.80)	3.90	0.06	0.02*
RSES	30.74 (4.95)	31.70 (5.41)	32.77 (4.68)	1.17	0.02	0.31
MPS self-oriented	68.09 (15.67)	74.98 (15.35)^c^	62.14 (11.61)	6.78	0.10	0.02*
MPS other-oriented	57.17 (12.52)	60.87 (9.73)	59.91 (9.20)	1.52	0.02	0.22
MPS socially prescribed	50.40 (12.97)	54 (13.31)	50 (8.16)	1.41	0.02	0.25
SPS total score	16 (10.57)	14.18 (9.64)	12.41 (7.73)	1.05	0.02	0.35
ORTO-15 total score	34.31 (3.54)	35.80 (3.44)	35.54 (4.07)	2.24	0.03	0.11

Note. MDDI = Muscle Dysmorphic Disorder Inventory; RSES = Rosenberg Self-Esteem Scale; MPS = Multidimensional Perfectionism Scale; SPS = Social Phobia Scale.

*p < 0.05. **p < 0.001.

^a^Significantly different to SA and FP; ^b^Significantly different to FP; ^c^Significantly different to FP.

### Psychological Features Related to MD Among Groups

Significant differences among groups emerged with respect to the MPS self-oriented subscale. Specifically, the SA group scored significantly higher than the FP group (*p* = 0.002), whereas the BB group did not differ from both the SA (*p* = 0.07) and the FP (*p* = 0.39) groups. Groups did not differ on the RSES (*p* = 0.31), MPS other-oriented scale (*p* = 0.22), MPS socially prescribed scale (*p* = 0.25), SPS (*p* = 0.35), and ORTO-15 (*p* = 0.11). Means, standard deviations, and comparisons are reported in Table 2.

### Psychological Features as Predictors of MD

#### BB group

In light of correlation findings, the RSES, the MPS socially prescribed subscale, the SPS, and the ORTO-15 were included in the second step of the two-steps hierarchical multiple regression. The overall model explained 49.1% of the variance in the MDDI total score. The DASS-21 total score was entered in the first step and significantly predicted the MDDI total score, *F*(1,40) = 6.62, *p* = 0.01, explaining 14% of the variation in the MDDI total score. The inclusion of the other variables in the second step explained an additional 41% of variation in the MDDI total score (*F* change = 8.27; *p* < 0.001). Results showed that, after controlling for the DASS-21 total score, the SPS and the ORTO-15 were significant predictors (*β* = 0.51 t = 3.95, *p* < 0.001 and *β* = −0.24 t = −2.12, *p* = 0.04, respectively), whereas the RSES (*p* = 0.06) and the MPS socially prescribed (*p* = 0.92) subscales were not.

#### SA group

The RSES and the SPS were included in the second step of the two-step hierarchical multiple regression. The overall model explained 23.3% of the variance in the MDDI total score. Results showed that, at step one, the DASS-21 total score was a significant predictor, *F*(1,59) = 9.22, *p* = 0.004, and accounted for 13% of the variation in the MDDI total score. Entering the other variables in step 2 explained an additional 14% of variation in the MDDI total score (*F* change = 0.14; *p* = 0.01). Results indicated that, after controlling for the DASS-21 total score, only the SPS was a significant predictor of the MDDI total score (*β* = 0.37 t = 2.83, *p* = 0.01), while the RSES was not (*p* = 0.39).

#### FP group

The RSES and the SPS were included in the second step of the two-step hierarchical multiple regression. The overall model explained 40.4% of the variance in the MDDI total score. Results showed that, at step one, the DASS-21 total score was a significant predictor, *F*(1,20) = 14.22, *p* = 0.001, and accounted for 42% of the variation in the MDDI total score. Entering the other variables in step 2 did not explain additional variance in the MDDI total score (*F* change = 1.29; *p* = 0.30).

## Discussion

MD is a subtype of BDD^1^ that is still under-recognized and frequently left untreated, despite its associated unhealthy behaviours (such as rigid adherence to dietary regimes, excessive exercise and, frequently, AAS use), which negatively impacts quality of life and well-being^2,3^. The current study aimed at expanding extant knowledge about MD in athletes at increased risk and, specifically, in three groups of recreational athletes: bodybuilders (BB group), strength athletes (SA group), and fitness practitioners (FP group).

In line with our expectations, individuals in the BB group displayed more MD-related behaviours than those in the other two groups; specifically, the BB group reported considering taking AAS more frequently than both the SA group and the FP group. Furthermore, the total protein intake was >2 g proteins/kg/day only in the BB group. These findings are consistent with studies underlining that being involved in sports that reward building muscles for appearance-related purposes, such as bodybuilding, may expose athletes to a greater risk of engaging in unhealthy behaviours such as use of AAS^16,28^. These results are also in accordance with studies reporting strict adherence to a high-protein diet in bodybuilders^25,29^. Importantly, individuals in the BB group think about taking AAS and engage in high-protein diet possibly due to dissatisfaction with their body size and shape and the desire to be more muscular. Accordingly, individuals in the BB group reported more beliefs about being smaller and weaker than desired and wished to be more muscular than the other two groups. A similar result was found by Lantz *et al*.^10^, who observed that bodybuilders are more likely to report body size-symmetry concerns than strength athletes because they are primarily interested in lifting weights to develop a mesomorphic physique, defined by muscular shape and symmetry. Therefore, individuals in the BB group may be concerned about their body size and shape and desire to be more muscular because of the goals of the weight training activity they perform; they struggle to reach the ideal body shape and may be concerned about failing to achieve it.

The BB group also reported more general symptomatology related to MD than the FP group, whereas no differences between the BB group and the SA group emerged. These results are in contrast with the meta-analysis by Mitchell *et al*.^11^ outlining that bodybuilders show greater MD symptomatology than strength training athletes. Nonetheless, these results are consistent with studies reporting that athletes involved in resistance training in general usually show increased MD symptoms^17,30^; consequently, the SA group may represent an intermediate group between BB and FP with respect to MD general symptomatology. Both bodybuilders and strength trainers usually engage in intensive sessions of weight training with the aim of increasing muscles size and strength, and this is achieved through intensive exercise. Thus, susceptible individuals of both groups may experience negative emotions when deviating from daily exercise and may experience anxiety in social situations if the desired muscle size and strength are not achieved. On the contrary, fitness practitioners would be less vulnerable to MD general sympotmatology in light of the different goals pursued through their training activity.

Regarding MD-related psychological features, no differences among groups emerged with the exception of self-oriented perfectionistic traits. Indeed, the SA group reported to set higher standards for themselves than the FP group, whereas no differences between the BB group and the FP group emerged. These findings are partially in line with the study by Muller *et al*.^25^, suggesting the presence of higher perfectionistic traits in bodybuilders and strength training athletes than in contact or noncontact sports athletes. However, the lack of differences in perfectionistic traits between the BB group and both the SA and the FP group was unexpected as the majority of previous studies underlined that perfectionistic traits are more prevalent in bodybuilders than in other athletes^27,31^. However, it is noteworthy that such studies focused on both competitive and recreational bodybuilders, and it may be possible that perfectionistic traits characterize competitive bodybuilders rather than recreational ones; therefore, our different sample composition (only recreational athletes) might explain discrepancies in results.

The three groups were comparable also with respect to self-esteem, orthorexic behaviours, and social anxiety symptoms; these results are rather unexpected. In accordance with most of literature about MD, we hypothesized lower levels of self-esteem^27^ and more orthorexic behaviours^25,29^ in the BB group than in both the SA and the FP groups. Regarding self-esteem, current findings mirror those by Muller *et al*.^25^ who failed to detect differences in self-esteem between bodybuilders/weight lifters and contact and non-contact sports. Similarly, Blouin and Goldfield^27^ did not find any difference in self-esteem between bodybuilders and runners, but they observed lower levels of self-esteem in bodybuilders than in martial athletes. Taken as a whole, current data might suggest that low self-esteem does not characterize bodybuilders, at least when bodybuilding is practiced with recreational purposes. The same might apply to orthorexic behaviours: indeed, a strict adherence to rigid diet and the obsession for healthy food might be more relevant in athletes who compete^32^. Concerning social anxiety, Pickett *et al*.^14^ also reported that symptoms of anxiety and social physique anxiety in bodybuilders are lower than, or comparable to, those displayed by strength training athletes and physically active controls. This may be explained, once again, by the absence of competitive bodybuilders in our sample. Indeed, individuals characterized by MD symptoms usually experience social anxiety symptoms in situations in which they are afraid of receiving a negative evaluation by others regarding their body^33^ which is definitely more common during competitions compared to recreational situations.

Lastly, findings about possible MD psychological predictors among groups showed that, within the BB group, MD symptomatology was predicted only by orthorexic behaviours and social anxiety symptoms. Social anxiety symptoms emerged as the only predictor of MD symptomatology also in the SA group. Results about social anxiety symptoms are somewhat in line with findings by Chandler *et al*.^34^ who underlined that these symptoms may represent a motivational factor underling MD symptoms through negative reinforcement. The fear of negative evaluation by others, especially about one’s physique, is an aversive state; since the social ideal for the male body is a mesomorphic physique^35–37^, an acceptable way to decrease social anxiety is gaining muscle through lifting weights. Therefore, lifting weights decreases anxiety, thus leading to the reinforcement of the weight lifting behaviours^33^. This mechanism may be peculiar for both the BB group and the SA group, given that they are both characterized by intensive sessions of weight training with the aim of increasing muscles size and strength. Regarding orthorexic behaviours, the obsession for healthy food may contribute to MD development only in bodybuilders. This result is in accordance with studies affirming that the majority of individuals with MD present dysfunctional eating patterns characterized by rigid diet and by the avoidance of food when its caloric content is unknown (e.g., eating food in restaurant)^4^. In other words, the obsession for healthy food might act as a trigger fostering the engagement in MD symptoms (e.g., beliefs of being smaller and weaker than desired, or wishing to be more muscular) and MD-related behaviours (e.g., engaging in intensive sessions of weight training). Regarding the FP group, no psychological features related to MD resulted predictive for MD symptomatology; although these results should be interpreted with caution in light of the small sample size of the FP group, they are reasonable since the FP group is not considered at increased risk of developing MD symptoms.

To note, the current study is characterized by several limitations. First, the sample size was relatively small which likely reduces the generalizability of our conclusions. Related to this issue, the statistical power of the employed regression design could not guarantee a reliable detection of MD predictors; hence, regression findings should be interpreted with caution. Secondly, the online recruitment strategy we adopted under-sampled those without internet capability and prevented the opportunity of conducting face-to-face interviews of participants self-identified as recreational bodybuilders and recreational strength athletes. Lastly, the absence of psychometric information for the MPS is another limitation.

To conclude, despite the above-mentioned shortcomings, the present study represents one of the first attempts to assess behaviours, symptoms, and possible psychological predictors of MD among athletes who trained regularly for recreational purposes. Future studies overcoming these issues are highly recommended. Indeed, to date, the distinction between pathological versus non-pathological pursuit of hyper muscularity has been scarcely addressed. Overall, the present findings suggest that the pursuit of a lean and muscular physique in bodybuilders is not always associated with MD and related psychological features. Although the BB group reported more negative beliefs about being smaller and weaker than desired, or wished to be more muscular than the other two groups, bodybuilders (as a group) are not necessarily characterized by psychological features related to MD such as low self-esteem, high perfectionism, social anxiety symptoms, and dysfunctional eating patterns. Therefore, the pursuit of a lean and muscular physique that characterized bodybuilders is not a sufficient condition to develop MD. Unlike from athletes not characterized by MD symptoms, athletes and individuals with MD set a high standard for their ideal body that, perhaps, is far from their reach. Additionally, the perception of their body may be far from their actual body. Therefore, pursuing the fitness of one’s own body is not related to MD but, rather, it is how extreme an individual sets their ideal body, and the level of discrepancy between their ideal and current body, that may contribute to the development of MD.

The present findings also suggest that orthorexic behaviours may predict MD symptomatology in bodybuilders; similarly, social anxiety symptoms may predict MD symptomatology both in bodybuilders and strength athletes. This information is crucial because it may lead to a better understanding and recognition of MD. Improving the identification of predictors and risk factors for MD, as well as its associated psychological features, might facilitate the identification of at-risk trainers, as well as the development of prevention strategies and interventions. Furthermore, a deeper understanding of MD manifestation among athletes may help to distinguishing individuals participating in a healthy manner versus those participating in an unhealthy manner.

## Methods

### Participants

Participants were 125 males who trained regularly for recreational purposes; they self-identified themselves as recreational bodybuilders (*n* = 42; BB group), recreational strength athletes (*n* = 61; SA group), and fitness practitioners (*n* = 22; FP group). All participants satisfied the following general inclusion criteria: at least 18 years of age; absence of severe neurological or medical conditions; no current or past psychotic disorders; no evidence of intellectual disabilities.

### Measures

All participants completed an online brief schedule collecting socio-demographic information, self-reported weight and height, daily protein intake, AAS use, and Pope *et al*.^2^ diagnostic criteria for MD. Furthermore, participants completed the following online self-report measures:

The *Muscle Dysmorphic Disorder Inventory* (MDDI^38^; Italian version by Santarnecchi & Dèttore, 2012^39^) is a 13 item self-report questionnaire assessing symptoms associated with muscle dysmorphia (MD) on a five-point Likert scale (from 0 = *never* to 4 = *always*). It contains three subscales: desire for size (DFS), appearance intolerance (AI), and functional impairment (FI); a total score can also be computed. The DFS subscale assesses beliefs of being smaller and weaker than desired, or wishing to be more muscular. The AI is made up of questions regarding negative beliefs and anxiety associated with one’s body and appearance. Lastly, the FI assesses the presence of negative emotions when deviating from daily exercise or avoidance of social situations because of the preoccupation with one’s body. The questionnaire showed good internal consistency, with Cronbach’s alpha coefficients raining from α = 0.77 to α = 0.85^38^. The Italian version proved to be highly reliable as well, with the exception of the AI subscale (α = 0.45)^39^. In the current study, the alpha coefficient was 0.80 for the DFS scale, 0.73 for the AI scale and 0.83 for the FI subscale. Finally, the alpha coefficient of the total score was 0.83.

The *Rosenberg Self-Esteem Scale* (RSES^40^; Italian version by Prezza, Trombaccia, & Armento, 1997^41^) consists of 10 items measuring global self-esteem. Items are rated on a four-point Likert scale, ranging from 1 = *strongly disagree* to 4 = *strongly agree*, with higher scores representing greater self-esteem. Good internal consistency values have been reported for the original RSES, ranging between α = 0.77 and α = 0.88^42^. The Italian version showed good psychometric properties as well: its internal consistency was α = 0.84^41^. The internal consistency coefficient was excellent also in the present sample (α = 0.88).

The *Multidimensional Perfectionism Scale* (MPS)^21^ is a 45 item self-report questionnaire designed to assess three different domains of perfectionism: self-oriented, socially prescribed, and other-oriented. The self-oriented subscale involves self-directed perfectionistic behaviours such as setting high standards for oneself; the socially prescribed subscale involves the perceived need to attain standards prescribed by significant others; and the other-oriented subscale involves placing unrealistic standards for significant others and placing importance on other people being perfect. Each subscale of the questionnaire contains 15 item evaluated on a seven-point Likert scale (ranging from 1 = *strongly disagree* to 7 = *strongly agree*), with higher scores indicating greater levels of perfectionism. With respect to the original MPS, good internal consistency values have been reported, ranging between α = 0.79 and α = 0.89 in a student sample^21^. The Italian validation of the MPS is not available to date, therefore an *ad hoc* translation was employed. In the present sample, the alpha coefficient was α = 0.89 for the self-oriented scale, α = 0.80 for the socially prescribed scale and α = 0.72 for the other-oriented perfectionism subscale.

The *Social Phobia Scale* (SPS^43^; Italian version by Sica *et al*.^44^) consists of 20 item assessing situations that involve being observed by others (e. g., public speaking, eating in public, etc.). Items are rated on a five-point Likert scale, ranging from 0 = *not at all* to 4 = *extremely*, with higher scores indicating greater social phobia. The SPS showed good psychometric properties; its internal consistency was α = 0.90 in a community sample and α = 0.89 in patients with social phobia. Test-retest reliability was *r* = 0.91 on a 4 weeks period^43^. The Italian version proved to be highly reliable and stable as well (internal consistency: α = 0.87; 30-day test-retest reliability: *r* = 0.87)^44^. Internal consistency coefficient was excellent in the present sample (α = 0.89).

The *ORTO-15*^45^ is a 15 item self-report questionnaire assessing orthorexia. The questionnaire evaluates the presence of obsessive attitudes towards choice, preparation and consumption of healthy foods on a four-point Likert scale ranging from 1 = *never* to 4 = *always*, with lower scores indicating orthorexic behaviours. Cut-off point values can be set depending on the purpose of the study^45^. Within the validation sample, the ORTO-15 demonstrated high specificity (73.6%) and high negative predictive value (100%) with a threshold value of 40 points^45^.

The *Depression Anxiety Stress Scale-21* (DASS-21^46^; Italian version by Bottesi *et al*.)^47^ is a 21 item self-report questionnaire assessing depression, anxiety, and stress on a four-point Likert scale (ranging from 0 = *did not apply to me at all* to 3 = *applied to me very much*), with higher scores indicating greater distress. Three subscale scores and a “general distress” total score can be computed^47^. The original DASS-21 demonstrated adequate reliability in non-clinical samples, with coefficient alphas ranging from α = 0.73 to α = 0.81^46^. The Italian version proved to be highly reliable as well, with internal consistency values ranging from α = 0.74 to α = 0.90 in a community sample^47^. For the purpose of the present research we focused only on the total score of the questionnaire. In the present study, the alpha coefficient for the total DASS-21 was excellent (α = 0.91).

### Procedure

Bodybuilders and strength athletes were recruited online through links posted on bodybuilding and resistance training discussion forums (www.projectinvictus.it; www.ironmanager.it); fitness practitioners were recruited in public gyms and were invited to complete online self-report questionnaires. All participants self-identified themselves as recreational bodybuilders, recreational strength athletes, and fitness practitioners. All individuals participated on a voluntary basis and provided their informed consent clicking agreement before starting to complete the survey about male body image and related psychological features; participants were also informed about the possibility to withdraw from the survey at any stage without explanation. After providing informed consent, participants completed the brief schedule collecting socio-demographic information and the self-report measures; responses were saved on the Google Drive server. Participants took approximately 30 minutes to complete the survey.

The study was conducted in accordance with the Declaration of Helsinki and approved by the Ethical Committee of the of the Psychological Sciences, University of Padova.

### Statistical Analyses

In order to assess group differences on socio-demographic variables, one-way analyses of variance (ANOVAs) and Chi-squared analyses were conducted. Bonferroni post hoc comparisons were computed when significant differences emerged.

Chi-squared analyses and one-way ANOVAs were performed in order to compare groups (BB group *vs* SA group *vs* FP group) regarding behaviours, symptoms, and psychological features related to MD; also in this case, Bonferroni post hoc comparisons were computed when appropriate.

In order to investigate MD psychological predictors we first calculated Pearson’s correlations taking into account the MDDI total score and possible psychological predictors (RSES, MPS, SPS, and ORTO-15) within each group (BB group, SA group, and FP group) (Table 3). Finally, based on correlation findings, we performed 3 two-step hierarchical regression analyses within each group (BB group, SA group, and FP group). The MDDI total score was the dependent variable and the DASS-21 total score was always included in the first block to control for general distress; finally, appropriated psychological variables were entered in the second block.

**Table 3:** Pearson’s correlations between MDDI total score and psychological features among groups.

	MDDI total score		
	BB group	SA group	FP group
RSES	−0.45**	−0.37**	0.64**
MPS self-oriented	0.10	0.18	−0.08
MPS other-oriented	−0.03	0.08	−0.01
MPS socially prescribed	0.39**	18	0.19
SPS total score	0.65***	0.48***	0.47*
ORTO-15 total score	−0.36*	−0.06	−0.03
DASS-21 total score	0.38**	0.37**	0.64**

Note. MDDI = Muscle Dysmorphic Disorder Inventory; RSES = Rosenberg Self-Esteem Scale; MPS = Multidimensional Perfectionism Scale; SPS = Social Phobia Scale.

*p < 0.05. **p < 0.01 ***p < 0.001.

Conventional significance levels were used (*ps* < 0.05). All statistical analyses were conducted using IBM SPSS statistics, version 21.

### Data availability

The datasets analysed during the current study are available from the corresponding author on reasonable request.
